# Identification of Key Genes Involved in Lactic Acid Metabolism in Periodontitis Based on Bioinformatics Analysis

**DOI:** 10.1111/jcmm.71141

**Published:** 2026-04-17

**Authors:** Bijun Zhu, Shi Xu, Mingyue Cheng, Leiying Miao

**Affiliations:** ^1^ Department of Cariology and Endodontics, Nanjing Stomatological Hospital, Affiliated Hospital of Medical School, Institute of Stomatology Nanjing University Nanjing Jiangsu China; ^2^ Department of Endodontics The Affiliated Stomatological Hospital of Nanjing Medical University Nanjing Jiangsu China

**Keywords:** immune infiltration analysis, lactic acid metabolism, machine learning, periodontitis

## Abstract

Lactic acid metabolism correlates with periodontitis progression. Using machine learning (LASSO and SVM‐RFE) on GSE16134 and GSE173078 datasets, *CFI* and *COQ2 were* identified as key genes, with good diagnostic performance (AUC > 0.75). GSEA linked these genes to MAPK signalling, endocytosis, and ubiquitin‐mediated proteolysis. Immune infiltration analysis revealed higher plasma cells and lower CD8 T cells in periodontitis. Molecular docking identified amiodarone as a potential compound with strong binding affinity to CFI and COQ2. The expression trends of *CFI* (upregulated) and *COQ2* (downregulated) were first confirmed in TNF‐α/IL‐1β‐stimulated human periodontal ligament cells and further validated in a rat ligature‐induced periodontitis model. These findings suggest that *CFI* and *COQ2* are potential diagnostic biomarkers and therapeutic targets for periodontitis.

## 1 Introduction

Periodontitis is a chronic inflammatory disease caused by dysbiosis of the oral microbiome and immune dysregulation. Currently recognized as the sixth most prevalent disease globally, it severely affects the health of nearly 10% of the world's population and imposes a substantial burden on healthcare expenditures [1]. The hallmark of periodontitis is the destruction of periodontal supporting tissues, characterized by clinical attachment loss, periodontal pocket formation, alveolar bone resorption, and tooth loss. Additionally, periodontitis has been linked to systemic conditions, including cardiovascular diseases [2] and diabetes [3]. Early clinical symptoms of periodontitis, such as gingival bleeding and halitosis, are often subtle, leading to delayed patient consultation. Traditional diagnostic methods, relying on periodontal probing depth and radiographic imaging, lack sensitivity and fail to accurately reflect disease activity [4]. Therefore, identifying key molecular biomarkers associated with the pathogenesis of periodontitis is critical for developing novel diagnostic tools and targeted therapies.

The pathogenesis of periodontitis is primarily attributed to the colonization of specific pathogenic microorganisms in dental plaque and dysregulated host immune responses. However, recent studies suggest that metabolic disturbances, including alterations in lactate, cholesterol, and D‐glucose levels, may contribute to disease progression, as evidenced by distinct metabolic profiles between gingivitis patients and healthy controls [5]. This implies that microenvironmental metabolic shifts may play a pivotal role in disease advancement. Lactate, the end product of the glycolytic pathway, was traditionally regarded as a metabolic waste. Emerging evidence, however, highlights its multifaceted roles as an energy substrate, signalling molecule, and immunomodulator, participating in cellular metabolic reprogramming, angiogenesis, inflammatory responses, and immune evasion [6]. A novel role of lactate is protein lactylation, which has recently emerged as a post‐translational modification of proteins to regulate gene expression [7]. For instance, lactate has been implicated in tumour progression and inflammatory diseases, yet its association with periodontitis remains unclear. The role of lactate in inflammatory diseases is complex and context‐dependent. On one hand, lactate enhances HIF‐1α stability, promoting the secretion of pro‐inflammatory cytokines such as IL‐1β and TNF‐α [8]. Conversely, lactate suppresses NF‐κB signalling via GPR81 receptor binding [9, 10, 11], or modulates anti‐inflammatory factor secretion through lactylation modifications [12]. A study has reported decreased lactate production and lactylation levels in the periodontal tissues of periodontitis rats and LPS‐stimulated human PDLSCs [13]. These findings suggest that lactate metabolism‐related genes may influence periodontitis progression, though their precise regulatory networks require further exploration.

**FIGURE 1:**
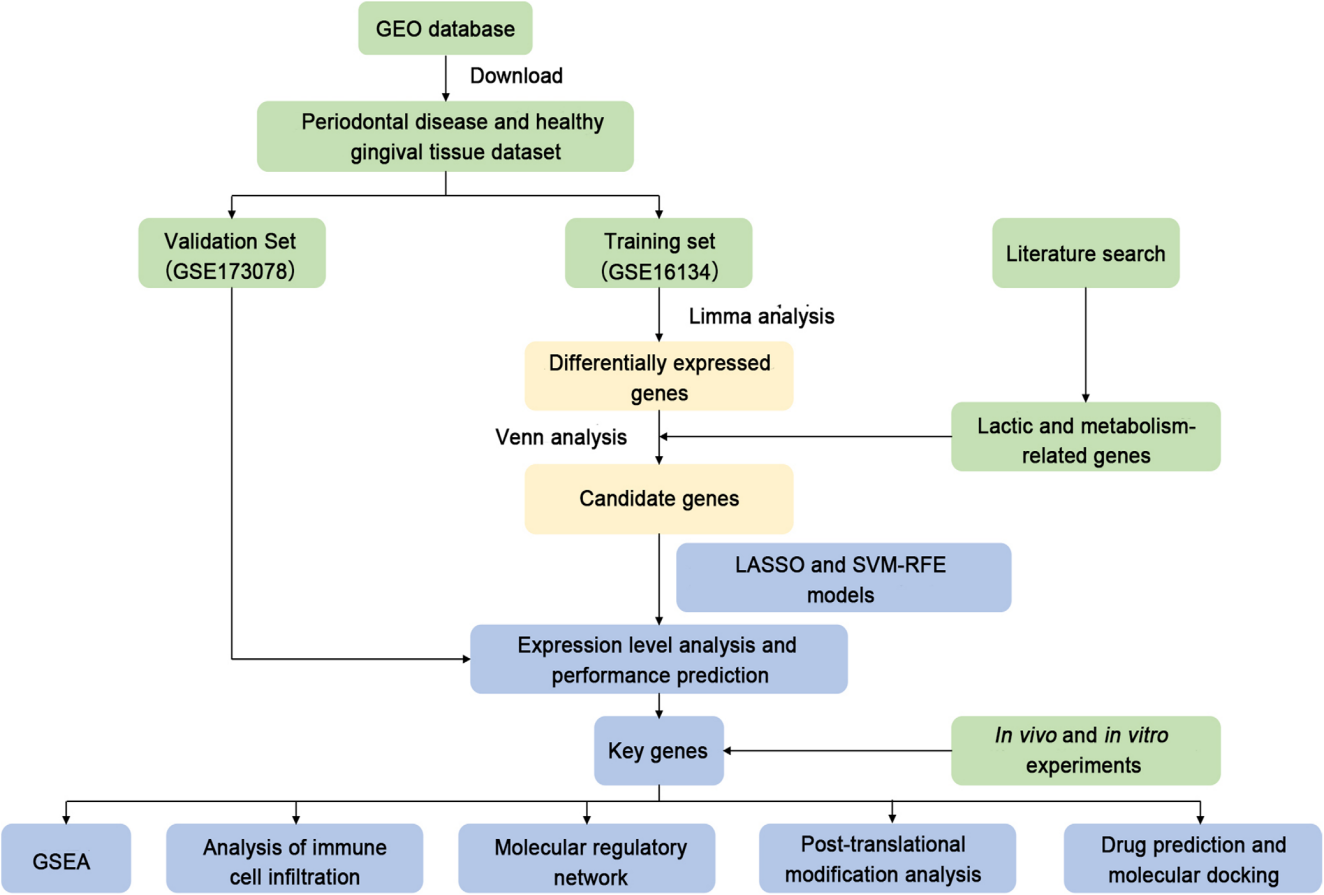
Flowchart of this study. Training and validation datasets were screened from public databases. Multiple bioinformatic analyses—including enrichment analysis, immune cell infiltration assessment, and molecular regulatory network construction—were integrated to identify and validate key genes. The study culminated in in vivo validation of differentially expressed genes (DEGs).

This study leverages public datasets to identify lactate metabolism‐related differentially expressed genes (DEGs) through differential expression analysis. Key genes are validated using machine learning, expression validation, and receiver operating characteristic (ROC) curve analysis. Subsequent functional enrichment analysis, immune infiltration profiling, molecular network construction, drug prediction, and molecular docking are performed to elucidate the molecular mechanisms of these key genes. Finally, the expression of DEGs was validated in vivo. The findings aim to provide novel insights for the diagnosis and treatment of periodontitis (Figure 1).

## 2 Material and Methods

### 2.1 Data Collection

The datasets relevant to periodontitis were retrieved from the Gene Expression Omnibus (GEO) database (http://www.ncbi.nlm.nih.gov/geo/). Among them, the dataset GSE16134 (platform GPL570) encompassed gingival tissue samples from 241 periodontitis and 69 control, which was designated as a training set. The validation set GSE173078 (platform GPL20301) encompassed gingival tissue samples from 12 periodontitis and 12 control (12 of the gingivitis samples were excluded). Furthermore, a total of 273 lactic acid metabolism‐related genes (LAMRGs) were obtained from the database (Supplementary Table 1).

### 2.2 Differential Expression Analysis and Identification of Candidate Genes

To identify differentially expressed genes (DEGs) between periodontitis and control samples within the training set, the ‘limma’ package (v 3.54.0) [14] was used for differential expression analysis, and DEGs were screened based on *p* < 0.05 and |log_2_ fold change (FC)| > 0.5. Subsequently, the ‘ggplot2’ package (v 3.4.1) [15] was utilized to construct a volcano plot for visualizing the DEGs, and the ‘pheatmap’ package (v 1.0.12) [16] was used to generate a heatmap of DEGs. To identify the DEGs linked to lactic acid metabolism in periodontitis, the ‘ggvenn’ package (v 0.1.9) [17] was used to intersect the DEGs and LAMRGs, and the intersection genes were used as candidate genes.

### 2.3 Identification and Validation of Key Genes

In the training set, in light of the candidate genes obtained above, the candidate feature genes were first screened using the least absolute shrinkage and selection operator (LASSO) regression analysis with 10‐fold cross‐validation via the ‘glmnet’ package (v 4.1.7) [18]. The optimal penalty parameter *lambda* was selected based on the minimum binomial deviance criterion, and genes with non‐zero coefficients at the optimal *lambda* were considered candidate features. Furthermore, the candidate feature genes were also screened using the support vector machine recursive feature elimination (SVM‐RFE) of the ‘e1071’ package (v 1.7.13) [19]. A linear kernel function was applied, and features were recursively eliminated with a step size of 1 to obtain the optimal feature subset based on the highest accuracy across cross‐validation folds. Then the Venn diagram was constructed using the ‘ggvenn’ package (v 0.1.9), and the genes obtained by the 2 algorithms were crossed and used as feature genes.

To observe whether the expression and trend of the feature genes were concordant in both the validation set and training set, the expression levels of the feature genes in the periodontitis and control samples were compared by Wilcoxon test in the 2 datasets (*p* < 0.05). Subsequently, the genes whose expression levels differed in the different samples and that had a consistent trend in the 2 datasets were selected and named as the candidate key genes.

To evaluate the ability of candidate key genes to discriminate periodontitis and control samples in different datasets, the ‘pROC’ package (v 1.18.0) [20] was utilized to plot the receiver operating characteristic (ROC) curves in the 2 datasets, and the area under the curve (AUC) was computed. Moreover, genes with an AUC value greater than 0.7 in both of the 2 datasets were named key genes for subsequent analyses.

### 2.4 Gene Set Enrichment Analysis (GSEA)

To access the functional pathways related to key genes in periodontitis, GSEA of key genes was performed. The reference gene set ‘c2.cp.kegg.v7.0.symbols.gmt’ was carefully chosen from the MSigDB. Firstly, based on all samples in the training set, Spearman correlation analyses were performed between each key gene and all the remaining genes separately using the ‘psych’ package (v 2.4.1) [21] to obtain the correlation coefficients (cor). Subsequently, genes were arranged in descending order according to these coefficients. The sorted data were then utilized to conduct GSEA (|normalized enrichment score (NES)| > 1 and *p* < 0.05) using the implementation of the ‘clusterProfiler’ package (v 4.2.2) [22].

### 2.5 Immune Infiltration Analysis

To understand the immune infiltration of different samples in the training set, the CIBERSORT algorithm (v 0.1.0) [23] was utilized to understand the distribution of 22 distinct immune cells [24] in both periodontitis and control samples. Subsequently, the Wilcoxon test was harnessed to investigate the disparities in the infiltration levels of these 22 immune cells between periodontitis and control (*p* < 0.05), and the outcomes were visualized using the ‘ggplot2’ package (v 3.4.1). Furthermore, Spearman correlation was carried out to investigate the connections between the differential immune cells and key genes (|cor| > 0.3 and *p* < 0.05) using the ‘cor’ package (v 1.18.3) [25].

### 2.6 Construction of Molecular Regulatory Network

To probe the intricacy and multiplicity of the regulation process of key genes' expression by constructing molecular regulatory network, the transcription factors (TFs) of key genes were forecasted using the JASPAR (https://jaspar.elixir.no/). Moreover, the miRNAs of key genes were forecasted using the ENCORI database (https://rnasysu.com/encori/). Finally, the TF‐mRNA and miRNA‐mRNA regulatory network was assembled by ‘Cytoscape’ software (v 3.8.2) [26].

### 2.7 Analysis of RNA‐Binding Protein (RBP) Network and Protein Post‐Translational Modifications

To understand the RBPs of key genes, the ENCORI database was also used for prediction, and the ‘Cytoscape’ software (v 3.8.2) was utilized to construct the regulatory network of key gene‐RBP.

To investigate the types of post‐translational modifications and related sites of key genes at the protein level, PhosphoSitePlus (https://www.phosphosite.org/homeAction.action) was used for analysis.

### 2.8 Compound Prediction and Molecular Docking

To probe the potential compounds for periodontitis and study potential associations between key genes and compounds, the Comparative Toxicogenomics Database (CTD, https://ctdbase.org/) was used to forecast compounds potentially connected with key genes, and ‘Cytoscape’ software (v 3.8.2) was used to render a visual representation of the compound‐key gene network.

To further validate the binding ability of compounds to key genes, molecular docking of each key gene to the corresponding compound was performed. The compounds shared by the key genes were selected, and after excluding the toxic and carcinogenic substances, the remaining compounds were subjected to molecular docking with the key genes. Initially, the 3D structure of compounds was extracted from the PubChem database. Subsequently, the 3D structure of the protein corresponding to the key genes was extracted from the RCSB Protein Data Bank. Thereafter, the docking simulation results were visualized using ‘MOE’ software (v 2022.02) [27].

### 2.9 Cell Culture

Human periodontal ligament cells (PDLCs) were purchased from ScienCell Research Laboratories (Carlsbad, CA, USA) and cultured in DMEM medium (Gibco, USA, C11995500BT) supplemented with 10% foetal bovine serum (Gibco, USA, 10099141) and 1% penicillin–streptomycin. For the assessment of osteogenesis‐related indicators, osteogenic components (β‐glycerophosphate, ascorbic acid, and dexamethasone) were additionally added to the culture medium.

### 2.10 Detection of Inflammatory Markers

For the detection of inflammation‐related markers, PDLCs were treated with DMEM medium containing 10 ng/mL TNF‐α and 10 ng/mL IL‐1β for 48 h based on references [28, 29], followed by RT‐qPCR experiments to measure the mRNA levels of the inflammatory factors IL‐1β, IL‐6, IL‐8, and TNF‐α. Total RNA was extracted using an RNA extraction kit (Vazyme, Nanjing, China). RNA was reverse‐transcribed into cDNA using the HiScript III RT SuperMix for qPCR (+gDNA wiper) reverse transcription kit (Vazyme, Nanjing, China). Using the primer sequences as templates, amplification was performed with a real‐time quantitative PCR instrument. The primer sequences are listed in Supplementary Table 2. The relative expression levels of the target genes were analyzed using the 2^^(‐ΔΔCt)^ relative quantification method.

### 2.11 Detection of Osteogenesis‐Related Indicators

After 7 days of osteogenic induction in PDLCs, the culture medium in the wells was removed, and the cell samples were washed 3–5 times with PBS for 3–5 min each time. Cells were stained using an Alkaline Phosphatase (ALP) Staining Kit (Beyotime, Shanghai, China). An appropriate amount of staining working solution was added, and the samples were incubated at room temperature protected from light for 30 min before images were captured. ALP activity was measured using an ALP Activity Assay Kit (Beyotime, Shanghai, China). Cells were lysed using cell lysis buffer, and ALP activity was calculated according to the kit instructions. mRNA levels of osteogenesis‐related markers were detected on day 14 of PDLCs culture, using the specific method described in section 2.10; primer sequences are listed in Supplementary Table 2.

After 21 days of osteogenic culture, calcium nodule formation was assessed in PDLCs. Specifically, cells were fixed with 4% paraformaldehyde for 15 min and stained with Alizarin Red S staining solution at room temperature for 30 min. Subsequently, 1 mL of 10% cetylpyridinium chloride was added to each well and fully dissolved at room temperature. The absorbance at 562 nm was measured using a microplate reader as a semi‐quantitative analysis result for calcium nodules.

### 2.12 In Vitro Validation of Key Genes and Their Protein Expression

After incubating PDLCs in medium with or without 10 ng/mL TNF‐α and 10 ng/mL IL‐1β for 48 h, the expression of *CFI* and *COQ2* was detected. The specific method was the same as that described in section 2.10, and the primer sequences are listed in Supplementary Table 2. Total protein was extracted, and the protein concentration was determined according to the instructions of the BCA Protein Assay Kit (Beyotime, Shanghai, China). Based on the protein concentrations measured by the BCA assay, different volumes of protein were loaded for electrophoresis. After protein separation, proteins were transferred to a PVDF membrane, which was then placed in blocking solution and blocked on a shaker at room temperature for 10 min. Subsequently, the membrane was incubated overnight at 4°C with the following primary antibodies: CFI (Abmart, Shanghai, China) and COQ2 (Bioss, Beijing, China), both diluted at a ratio of 1:1000. The membrane was washed three times with TBST on a shaker for 10 min each time. Afterwards, it was incubated with a secondary antibody at room temperature for 1 h, followed by exposure using a chemiluminescence imaging system. Band intensities were analysed using Image J software.

### 2.13 Construction of Rat Periodontitis Model and Validation of Key Gene Expression

All animal experiments were approved by the Animal Ethics Committee of Nanjing University (Approval No. IACUCD240258) and conducted in accordance with the National Institutes of Health Guide for the Care and Use of Laboratory Animals. Seven‐week‐old male Sprague–Dawley (SD) rats were selected and randomly divided into two groups: a control group and a ligation grouAfter one week of acclimatization feeding, the rats were anaesthetised. Under sterile conditions, the bilateral maxillary second molars of rats in the ligation group were ligated using sterilized 0.2 mm diameter orthodontic ligature wire. The control group had the wire immediately removed to exclude the influence of the ligation procedure. After 4 weeks of bilateral maxillary second molar ligation in rats, the animals were euthanized via cervical dislocation under excessive sodium pentobarbital anaesthesia, and maxillary bone samples were collected. Maxillary alveolar bone morphology and quality were scanned using micro‐CT (Bruker, Germany). Maxillary tomographic images were reconstructed using CTvox and DataViewer software. CTAn software was used to measure the distance from the cementoenamel junction (CEJ) to the alveolar crest at six periodontal sites per maxillary second molar. This distance, indicative of bone loss level, was statistically analysed to confirm the successful establishment of the rat periodontitis model. Immunohistochemistry was performed to detect the expression of CFI (Abmart, China) and COQ2 (Bioss, China) in the periodontal tissues surrounding the maxillary second molars.

### 2.14 Statistical Analysis

Bioinformatics analyses were conducted using the R programming language (v 4.2.2). The differences between the 2 groups were assessed by Wilcoxon test, with a significance level of *p* < 0.05 being deemed statistically significant.

## 3 Results

### 3.1 Identification of Candidate Genes Related to Lactic Acid Metabolism

**FIGURE 2:**
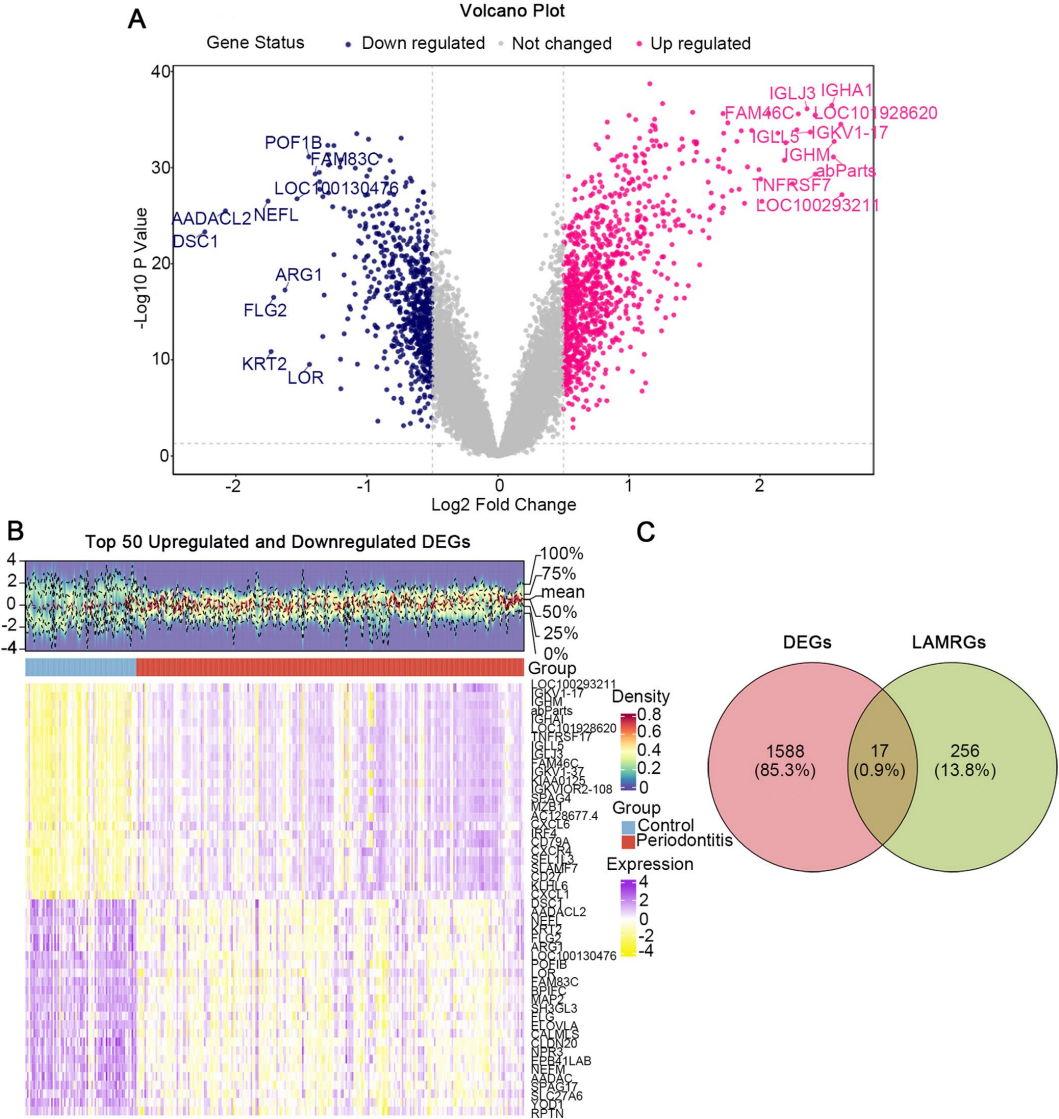
Identification and screening of differentially expressed genes (DEGs). (A) Volcano plot depicting DEG distribution between periodontitis and control groups. Down regulated genes are shown in blue, not changed genes in grey, and up regulated genes in red. (B) Heatmap of gene expression patterns displaying the top 50 DEGs from the GSE16134 dataset. (C) Venn diagram illustrating the overlap between DEGs and lactate metabolism‐related genes (LANRGs).

There were 1605 DEGs between the periodontitis and control samples in the training set, including 956 up‐regulated genes and 649 down‐regulated genes (Figure 2A,B). To further identify DEGs associated with lactic acid metabolism, 1605 DEGs and 273 LAMRGs were intersected, and 17 intersection genes were obtained as candidate genes (Figure 2C). Overall, the comprehensive analysis had offered a wealth of information that could guide further research efforts aimed at understanding the underlying mechanisms of periodontitis and developing innovative therapeutic strategies.

### 3.2 A Total of 2 Key Genes That Impact Periodontitis Were Discovered

**FIGURE 3:**
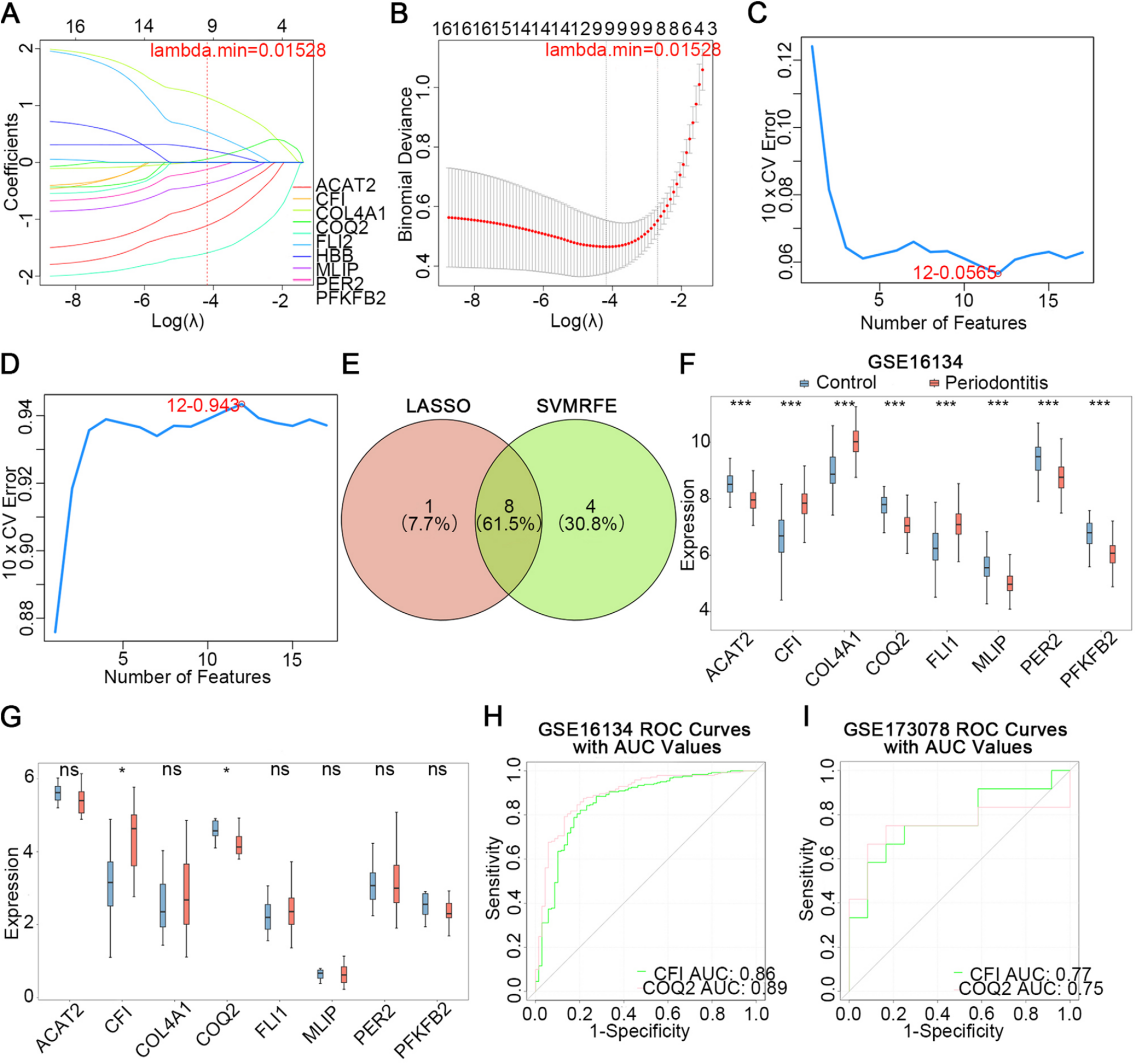
Machine learning‐based screening of candidate key genes *CFI* and *COQ2*. (A‐B) Least absolute shrinkage and selection operator (LASSO) algorithm identified 9 candidate feature genes, (A) Coefficient profile plot of LASSO regression; (B) Cross‐validation error curve (*lambda* selection). (C‐D) Support vector machine (SVM) analysis screened 12 candidate feature genes, (C) Error rate variation under 10‐fold cross‐validation; (D) Accuracy rate variation under 10‐fold cross‐validation. (E) Venn diagram showing 8 overlapping genes from both algorithms. (F) Expression analysis of feature genes in the training set. (G) Expression analysis of feature genes in the validation set. (H) Receiver operating characteristic (ROC) curves for *CFI* and *COQ2* in the training set. (I) ROC curves for *CFI* and *COQ2* in the validation set.

Initially, based on the candidate genes, 9 candidate feature genes were acquired by LASSO regression analysis with the *lambda*.min value of 0.01528 (Figure 3A,B). In addition, through the SVM‐RFE, a total of 12 candidate feature genes were obtained (Figure 3C,D). Finally, 8 intersection genes of the 2 algorithms (*ACAT2*, *CFI*, *COL4A1*, *COQ2*, *FLI1*, *MLIP*, *PER2*, and *PFKFB2*) were obtained, and they were used as feature genes (Figure 3E).

Moreover, in the training set, these 8 feature genes manifested pronounced disparities in expression between periodontitis and control samples (Figure 3F); while in the validation set, only the *CFI* and *COQ2* genes were significantly different between the periodontitis and control samples, and their expression trend was consistent with the training set (Figure 3G). Among them, *CFI* was upregulated in periodontitis and *COQ2* was downregulated in periodontitis compared with control. Therefore, *CFI* and *COQ2* were utilized as candidate key genes.

Furthermore, to assess the diagnostic utility of candidate key genes in periodontitis, the ROC curve analysis was conducted. Interestingly, it was found that the AUC values of candidate key genes (*CFI* and *COQ2*) were 0.86, 0.89 respectively (Figure 3H). In the validation set, the AUC values of candidate key genes (*CFI* and *COQ2*) were 0.77, 0.75 respectively (Figure 3I). These outcomes showed that the diagnostic efficacy of *CFI* and *COQ2* was satisfactory, and thus they could serve as key genes for subsequent analysis.

### 3.3 Key Genes Participated in the Regulation of Periodontitis Through Diverse Pathways and They Were Associated With Immune Cells

**FIGURE 4:**
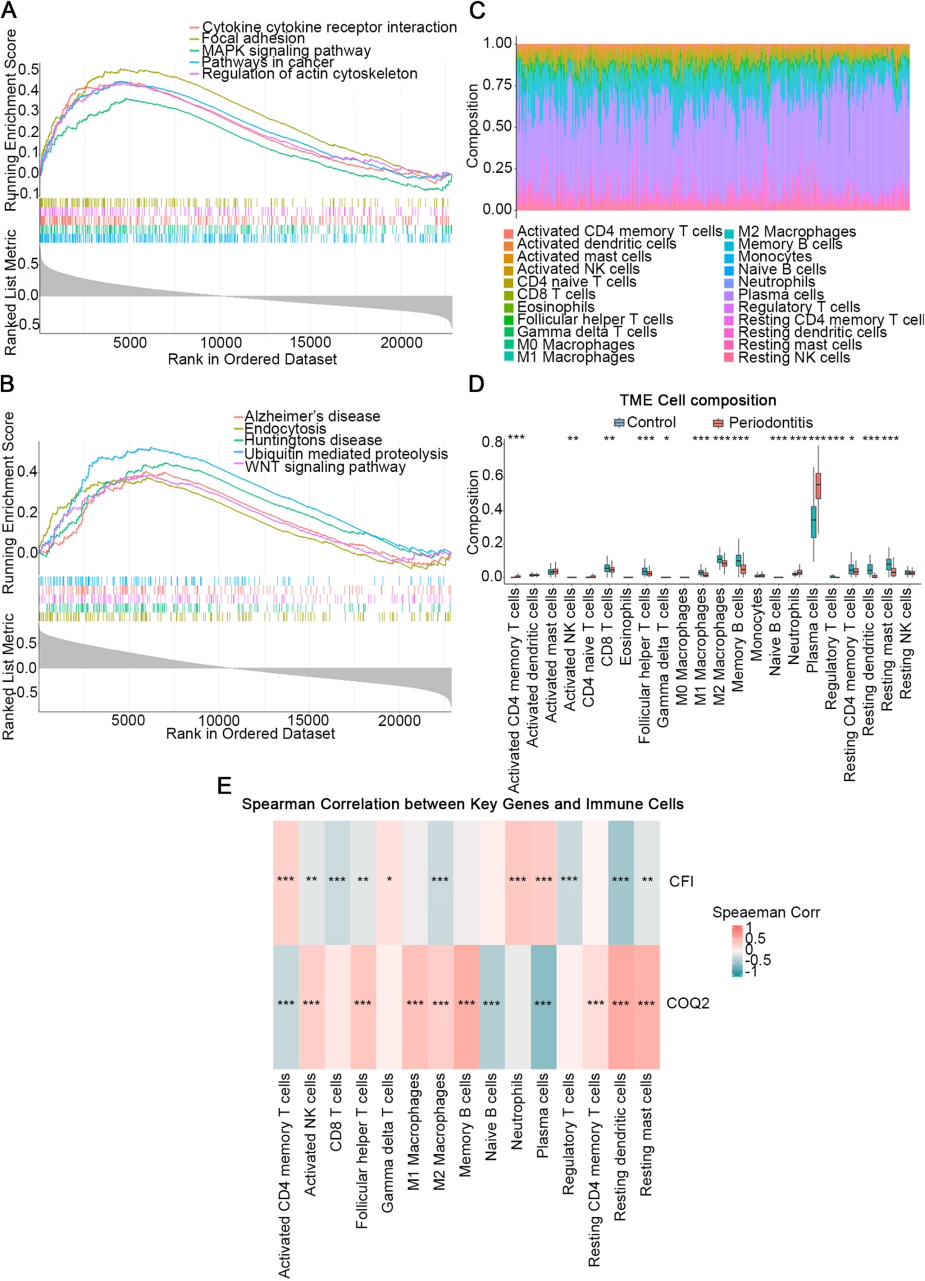
Functional profiling of key genes. (A) Gene Set Enrichment Analysis (GSEA) for *CFI* showing the top five enriched pathways ranked by normalized enrichment score (NES). (B) GSEA for *COQ2* displaying the top five enriched pathways based on NES. (C) Heatmap of infiltration levels for 22 immune cell types in healthy controls versus periodontitis samples. (D) Box plot comparing immune cell distributions between control (blue) and periodontitis (red) groups across 22 cell subtypes. (E) Correlation matrix of 15 differentially infiltrated immune cells, with negative correlations in blue and positive correlations in red. * means *p* < 0.05, ** means *p* < 0.01, *** means *p* < 0.001 relative to the Control group.

GSEA results for the 2 key genes showed that *CFI* was enriched in 106 pathways, and the top 5 pathways were MAPK signalling pathway, regulation of actin cytoskeleton, pathways in cancer, cytokine‐cytokine receptor interaction, and focal adhesion (Figure 4A, Supplementary Table 3); *COQ2* was enriched in 96 pathways, and the top 5 pathways were endocytosis, Huntingtons disease, Wnt signalling pathway, Alzheimer's disease, and ubiquitin mediated proteolysis (Figure 4B, Supplementary Table 4).

The proportional distribution of 22 immune cells in periodontitis and control samples was presented in Figure 4C. There were 15 differential immune cells, including activated NK cells, CD8 T cells, follicular helper T cells, M1 macrophages, M2 macrophages, etc. The outcomes revealed that compared with the control, the periodontitis samples had a higher proportion of plasma cells and a lower proportion of CD8 T cells (*p* < 0.05) (Figure 4D). Furthermore, *CFI* had the most notable positive relationship with neutrophils and the most notable negative relationship with resting dendritic cells (cor = 0.33, −0.48, *p* < 0.001), while COQ2 exhibited the most notable positive relationship with resting dendritic cells and the most notable negative relationship with plasma cells (cor = 0.57, −0.59, *p* < 0.001) (Figure 4E). The above results showed that key genes might affect the immune microenvironment of periodontitis patients, which could offer a valuable reference for the clinical decision‐making in the periodontitis treatment.

### 3.4 Multiple Molecules and Compounds Might Be Related to the Key Genes in Periodontitis

**FIGURE 5:**
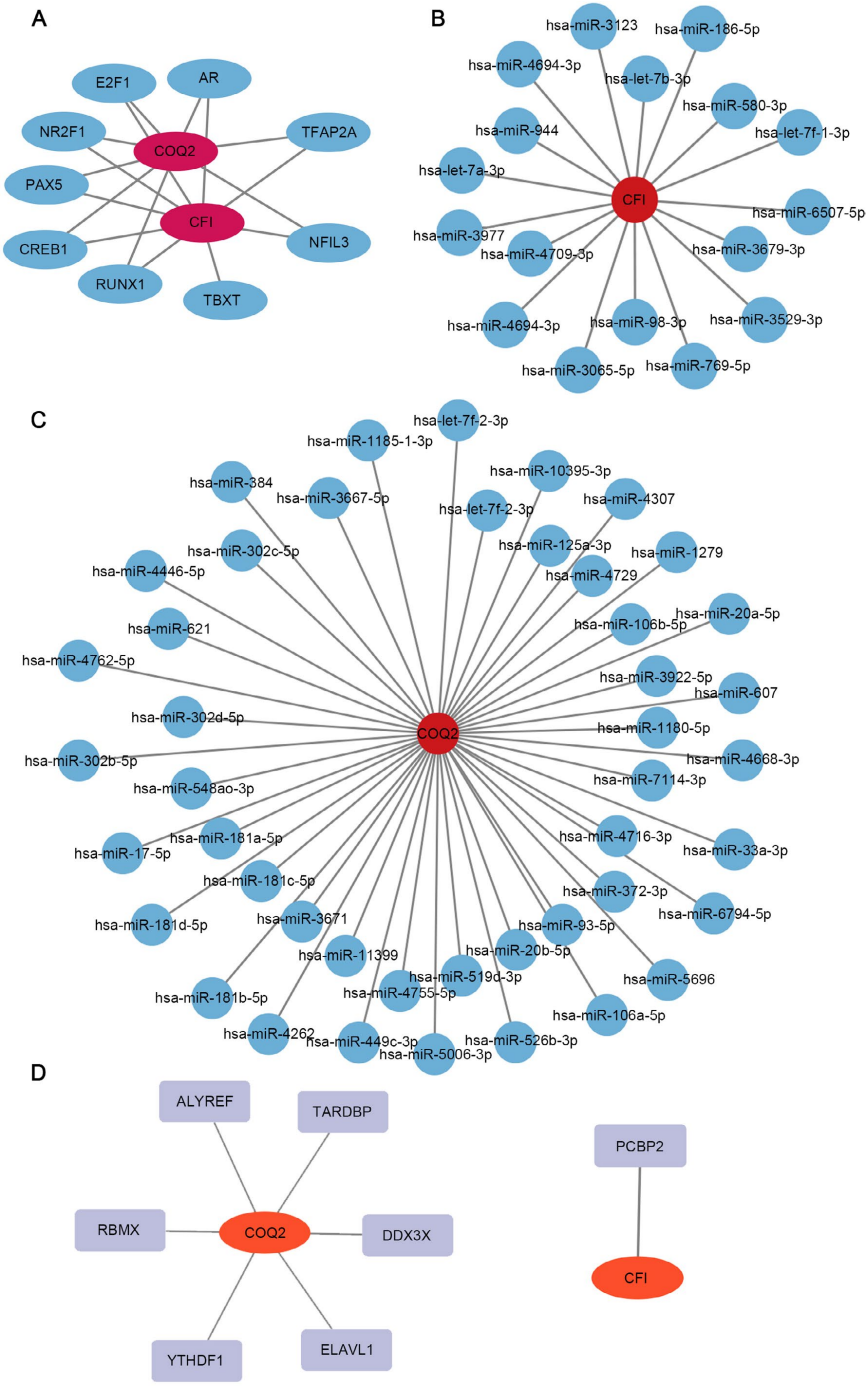
Molecular regulatory networks of key genes. (A) mRNA‐transcription factor (TF) regulatory network for *CFI* and *COQ2*. Red nodes denote the two key genes; blue nodes represent TFs. (B) Predicted mRNA‐miRNA interaction network for *CFI*. (C) Predicted mRNA‐miRNA interaction network for *COQ2*. Red nodes indicate key genes; blue nodes denote miRNAs. (D) mRNA‐RNA‐binding protein (RBP) interaction network. Red nodes mark key genes; purple nodes signify RBPs.

The results of the transcription factor regulatory network showed that a total of 9 TFs regulated the expression of key genes. Among them, 8 TFs jointly regulated the expression of both *CFI* and *COQ2*, such as TFAP2A, NFIL3, and AR (Figure 5A). A total of 17 miRNAs targeting *CFI* were predicted, such as hsa‐miR‐6759‐3p and hsa‐miR‐3679‐3p; 45 miRNAs targeting *COQ2* were predicted, such as hsa‐miR‐302b‐5p and hsa‐miR‐621 (Figure 5B,C). A total of 1 RBP interacting with *CFI* was identified, namely PCBP2. For *COQ2*, 6 interacting RBPs were found, including ALYREF, DDX3X, ELAVL1, RBMX, TARDBP, and YTHDF1. (Figure 5D).

**FIGURE 6:**
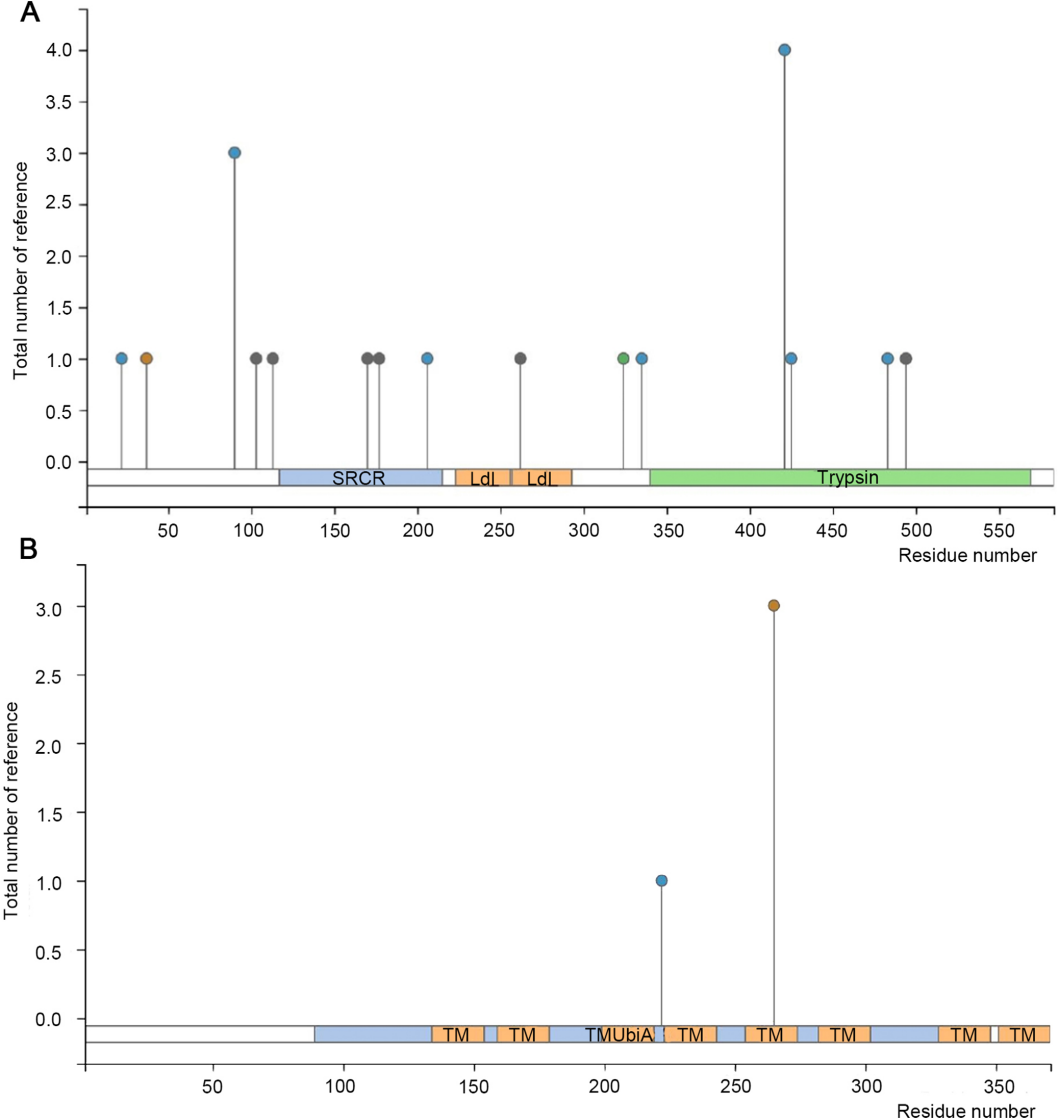
Post‐translational modification (PTM) analysis of key genes. (A) PTM types and associated residues of the CFI protein. (B) PTM types and associated residues of the COQ2 protein.

The findings regarding the post‐translational modifications of the proteins encoded by the 2 key genes indicated that CFI harboured 7 phosphorylation sites, 1 acetylation site, and 1 ubiquitination site (Figure 6A). COQ2 was found to have 1 phosphorylation site and 1 ubiquitination site (Figure 6B). These results not only facilitated a more in‐depth comprehension of the post‐translational modification profiles of the key gene‐derived proteins but also offered valuable references for the treatment of periodontitis.

**FIGURE 7:**
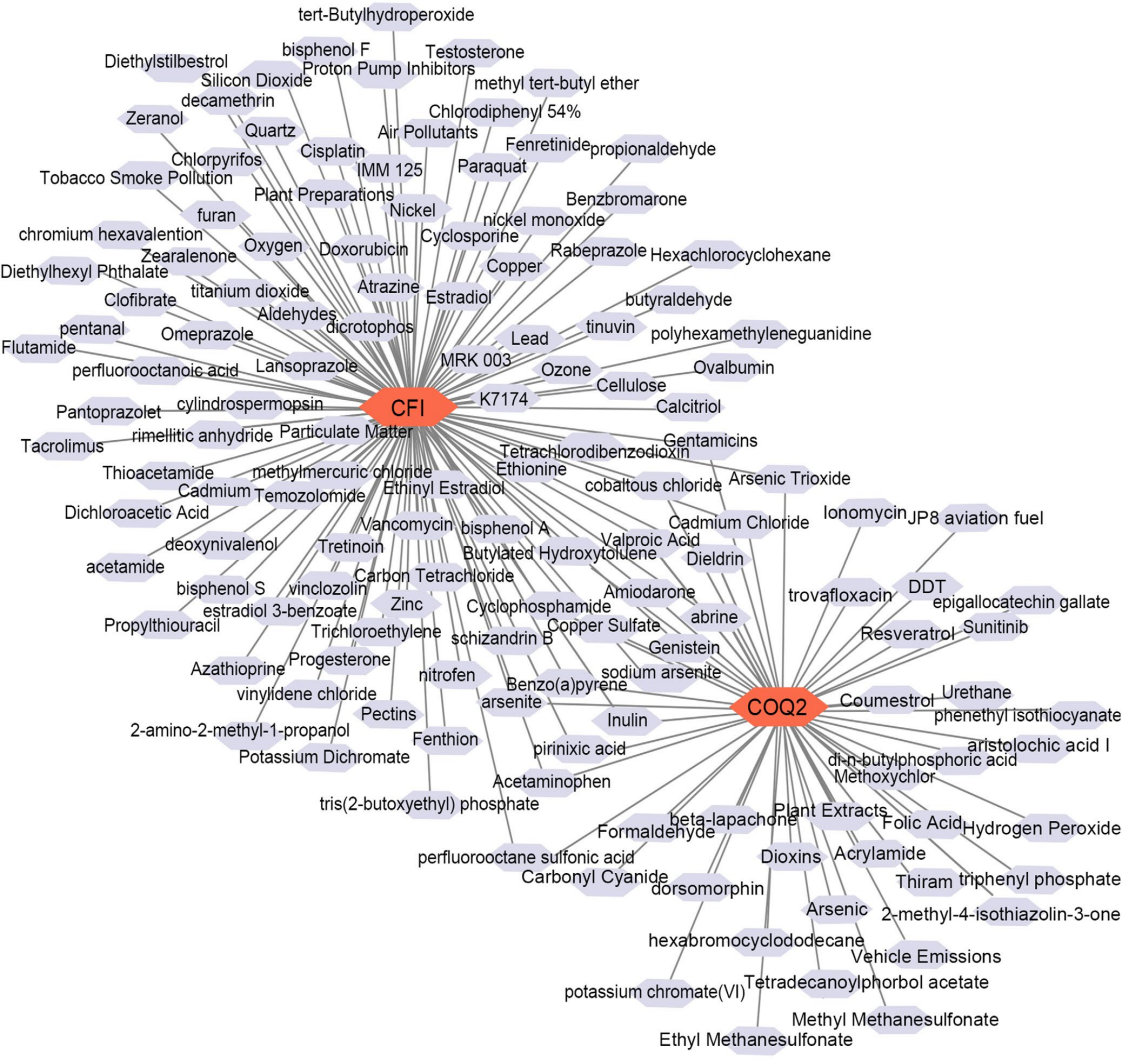
Drug‐gene targeting network. Red nodes represent key genes; purple nodes indicate predicted drugs.

**FIGURE 8:**
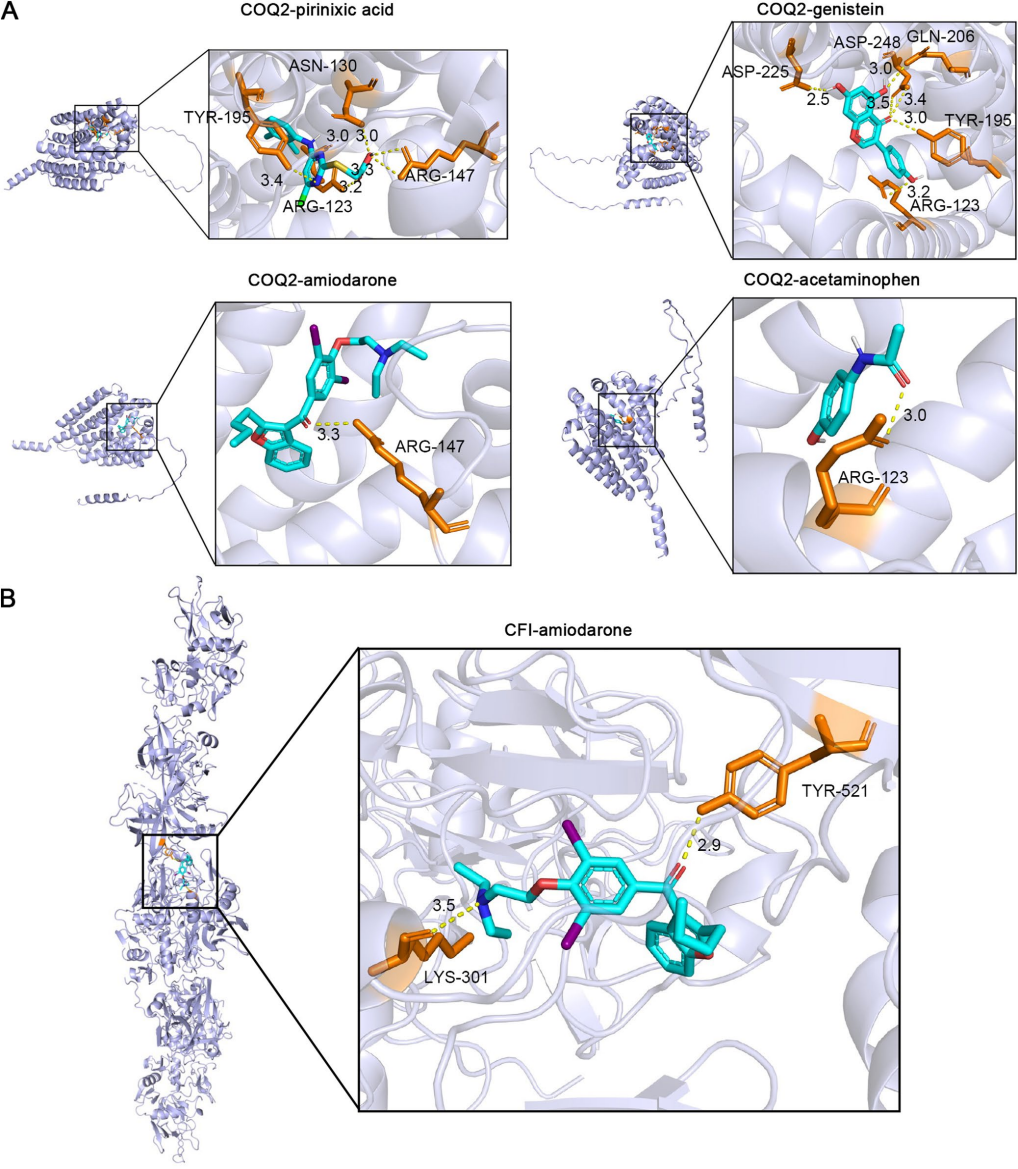
Molecular docking of key genes with drug candidates. (A) Docking conformations between COQ2 and small‐molecule ligands: Acetaminophen, pirinixic acid, amiodarone, and genistein. (B) Binding mode of CFI with Amiodarone.

Based on the CTD, the potential compounds of key genes were predicted. Among them, 120 compounds were predicted for *CFI*. In total, 55 compounds were forecasted for *COQ2* (Figure 7). A total of 20 common compounds were predicted for the 2 key genes, including abrine, acetaminophen, and amiodarone. After excluding the toxic and carcinogenic substances, a total of 4 common compounds were obtained, including acetaminophen, amiodarone, genistein, and pirinixic acid. The molecular docking outcomes demonstrated that the binding affinities of CFI and COQ2 with amiodarone were −6.71 and −7.27 (Table 1), respectively. In addition, the binding energies of genistein and pirinixic acid with COQ2 were −5.92 and −6.85, respectively, which were all less than −5 kcal/mol, signifying that the key genes possessed strong affinity with compounds. The analysis of binding conformation revealed that the compound infiltrated into the binding site of each key gene, and there were abundant hydrogen bond donors and acceptors around the binding sites (Figure 8A,B, Supplementary Figure 1). The association of key genes with other molecules and compounds could potentially offer a valuable reference for the clinical management of periodontitis.

**TABLE 1:** Molecular docking results of CFI and COQ2.

protein	PDB ID/AFold ID	intersection chem	Compound CID	S
CFI	2XRC	Acetaminophen	1983	/
		Amiodarone	2157	−6.713953
		Genistein	5,280,961	/
		pirinixic acid	5694	/
COQ2	AF‐Q96H96‐F1	Acetaminophen	1983	−4.84126234
		Amiodarone	2157	−7.27498102
		Genistein	5,280,961	−5.91755962
		pirinixic acid	5694	−6.85080624

### 3.5 In Vivo and in Vitro Validation of Key Gene Expression Changes

Given the observed changes in the expression of the *CFI* and *COQ2* genes within the dataset, and based on existing literature, we utilized a cytokine mixture of TNF‐α and IL‐1β to co‐culture with Human Periodontal Ligament Cells (PDLCs) to establish an in vitro model of periodontitis. This approach aimed to detect alterations in both the gene and protein levels of *CFI* and COQ2 at the cellular level.

**FIGURE 9:**
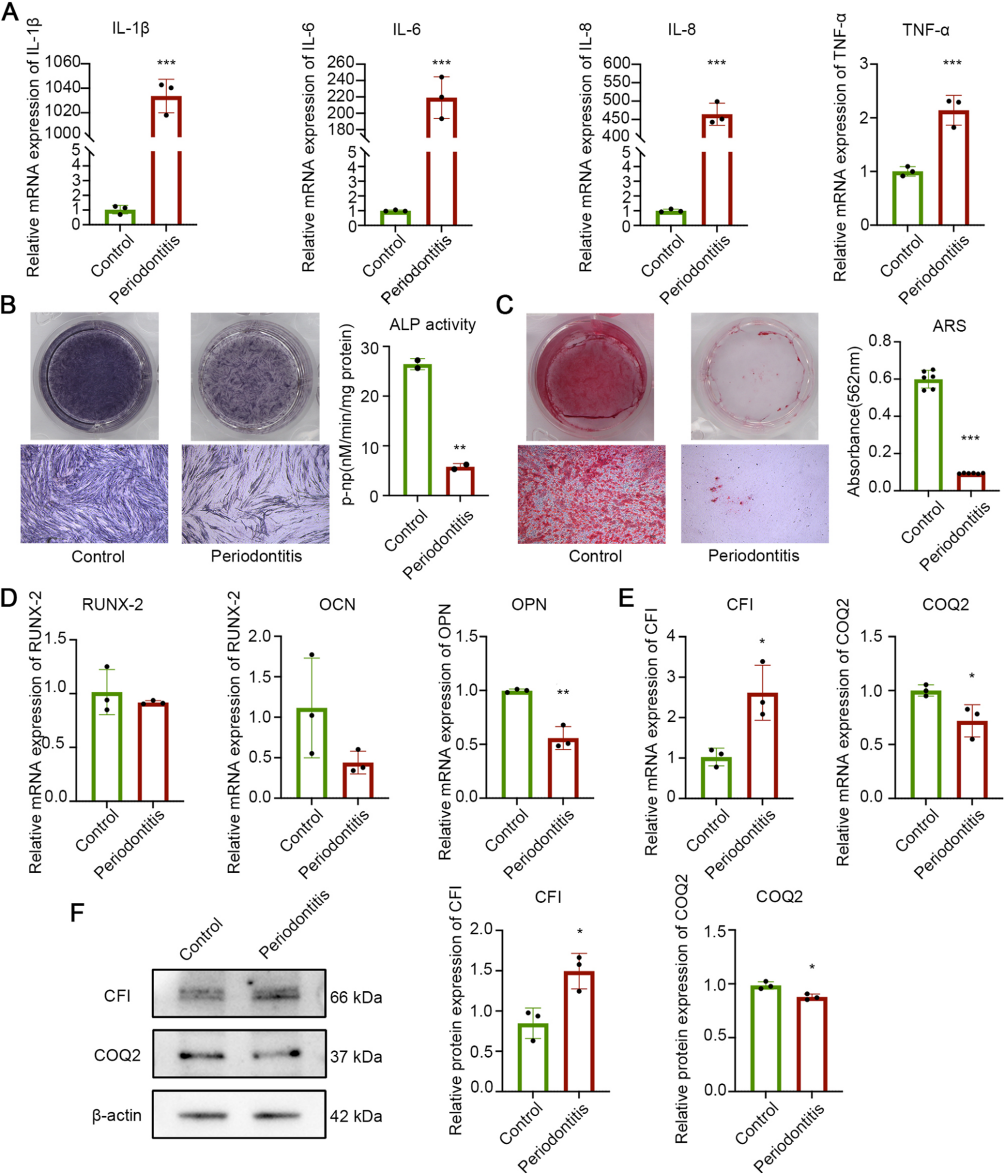
Changes in the mRNA and protein expression of *CFI* and *COQ2* were confirmed in the in vitro periodontal inflammation model. (A) mRNA levels of inflammation‐related cytokines (IL‐1β, IL‐6, IL‐8, TNF‐α) under inflammatory conditions. (B) Changes in Alkaline Phosphatase (ALP) activity in the periodontal inflammation model. The left panel shows representative images of ALP staining, and the right panel shows the quantitative results of ALP activity. (C) Calcium nodule formation in the periodontal inflammation model. The left panel shows representative images of Alizarin Red S (ARS) staining, and the right panel shows the semi‐quantitative analysis of ARS results. (D) mRNA expression levels of osteogenesis‐related factors (*RUNX‐2*, *OCN*, *OPN*). (E) mRNA expression levels of the key genes *CFI* and *COQ2*. (F) Protein expression levels of CFI and COQ2. * means *p* < 0.05, ** means *p* < 0.01, *** means *p* < 0.001 relative to the Control group.

After treating PDLCs with TNF‐α and IL‐1β, we first measured the mRNA levels of several hallmark inflammatory factors. The mRNA levels of these inflammatory factors were significantly increased (Figure 9A). Osteogenesis‐related indicators, including Alkaline Phosphatase (ALP) activity, the quantity of calcium nodule formation, and the mRNA levels of osteogenesis‐related factors, were all significantly decreased. These changes are consistent with the characteristics of periodontitis and confirmed the successful establishment of the model (Figure 9B–D).

RT‐qPCR results demonstrated that PDLCs in the inflammatory environment exhibited increased *CFI* expression and decreased *COQ2* expression (Figure 9E). Detection at the protein level similarly confirmed these findings. Under inflammatory conditions, the expression level of CFI was elevated, while the expression of COQ2 was reduced (Figure 9F). These discoveries verify that the mRNA and protein expression levels of *CFI* and *COQ2* in the periodontal inflammatory environment are consistent with the trends observed in the dataset.

**FIGURE 10:**
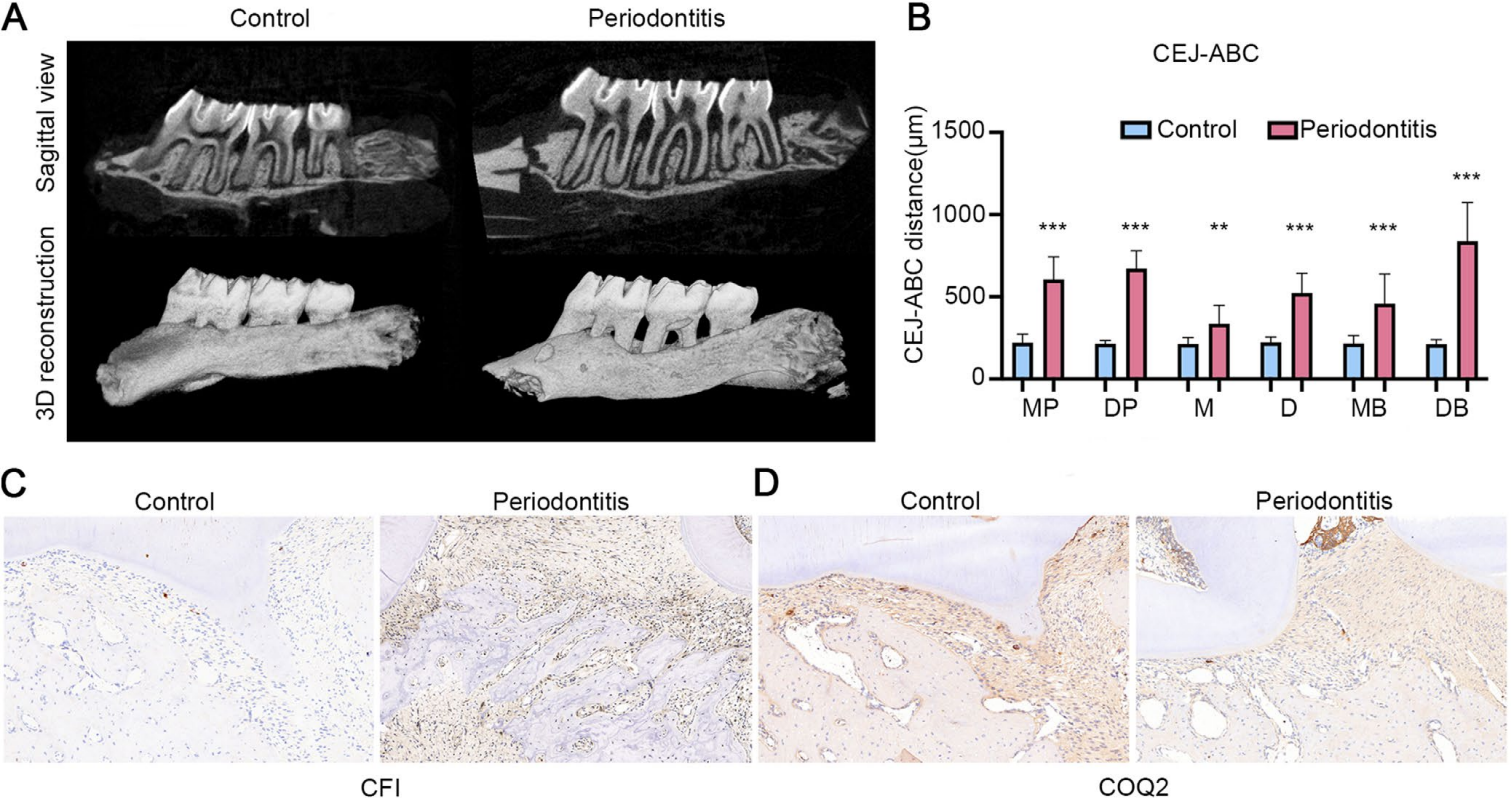
In vivo validation of key gene expression alterations in a rat ligature‐induced periodontitis model. (A) Micro‐CT analysis depicting sagittal sections and 3D reconstructions of maxillary second molars. (B) Cementoenamel junction‐to‐alveolar bone crest (CEJ‐ABC) distances at six periodontal sites: Mesiopalatal (MP), distopalatal (DP), mesial (M), distal (D), mesiobuccal (MB), and distobuccal (DB). (C) Immunohistochemical staining revealed increased CFI expression in periodontitis tissues. (D) Decreased COQ2 expression was observed in periodontitis tissues by immunohistochemistry. ** means *p* < 0.01, *** means *p* < 0.001 relative to the Control group.

To further validate the diagnostic value of key genes *CFI* and *COQ2*, we established a rat model of experimental periodontitis using the ligature‐induced method. Orthodontic ligature wires were placed around the maxillary second molars to induce periodontal inflammation. Gene expression was verified at both tissue and molecular levels. Micro‐CT analysis was first performed. Sagittal sectional and 3D‐reconstructed images revealed alveolar bone conditions, demonstrating reduced bone height surrounding the teeth, confirming successful periodontitis induction (Figure 10A). The cementoenamel junction‐to‐alveolar bone crest (CEJ‐ABC) distance was measured at six sites of the second molars: mesiopalatal (MP), distopalatal (DP), mesial (M), distal (D), mesiobuccal (MB), and distobuccal (DB). Significant increases in CEJ‐ABC distances in the periodontitis group validated alveolar bone loss in the rat model (Figure 10B). Immunohistochemical staining showed elevated expression of CFI (Figure 10C) and reduced expression of COQ2 (Figure 10D) in periodontitis‐induced rats compared to healthy controls.

## 4 Discussion

Periodontitis is a chronic inflammatory disease associated with systemic disorders. Recent studies suggest potential links between metabolic dysregulation and periodontitis [30]. Lactate metabolism plays a complex, dual role in the pathogenesis of periodontitis. This study integrated multi‐level bioinformatics analyses and machine learning to identify *CFI* and *COQ2* as key genes bridging lactate metabolism and periodontitis progression. These findings expand the understanding of periodontal pathogenesis and strengthen the connection between periodontitis and metabolic research. Compound‐binding predictions for differential genes reveal potential therapeutic agents, contributing to the advancement of precision medicine in periodontitis treatment.

The data sources for this study include two datasets: the training set GSE16134 and the validation set GSE173078, used for differential expression analysis and external validation. The training set GSE16134 contains 241 affected gingival tissue samples from periodontitis patients and 69 unaffected gingival tissue samples from the same patients (control samples from healthy gingival tissues), significantly reducing inter‐individual heterogeneity and improving the accuracy of genetic detection. Differential expression analysis was performed on the training set samples. Genes related to lactate metabolism were intersected from the obtained DEGs. Subsequently, two independent machine learning methods, LASSO and SVM‐RFE models, were used to further screen the identified feature genes. Combined with the validation set GSE173078, external validation of candidate key genes was conducted, yielding the key genes *CFI* and *COQ2*. *CFI* and *COQ2* exhibited AUC > 0.7 in both datasets, indicating their high predictive ability for periodontitis.

The *CFI* gene encodes complement factor I, a serine protease that cleaves and inactivates complement components C4b and C3b, thereby preventing the assembly of C3 and C5 convertases, which is critical for regulating the complement cascade. Recent studies have shown elevated expression of C3, C3b, and C4b in gingival tissues of periodontitis patients, with their levels positively correlated to disease severity. Inhibitors of C3b/C4b have been shown to mitigate alveolar bone loss in periodontitis [31, 32]. This suggests that the upregulation of *CFI* observed in periodontitis groups in this study may reflect a host defence response, where *CFI* likely modulates C3b and C4b to influence disease progression. In terms of lactate metabolism, lactate activates the GPR81 receptor, promoting M2 macrophage polarization, which may indirectly affect the CFI‐associated complement system. The *COQ2* gene encodes 4‐hydroxybenzoate polyprenyltransferase, an enzyme that catalyses the second critical step in coenzyme Q (CoQ) biosynthesis. CoQ is essential for mitochondrial energy metabolism and antioxidant defence [33, 34]. The role of *COQ2* in periodontitis may involve mitochondrial dysfunction, oxidative stress, and inflammatory regulation. In this study, *COQ2* was found to be downregulated in periodontitis groups, leading to reduced mitochondrial oxidative phosphorylation efficiency, increased mitochondrial reactive oxygen species (ROS), and impaired oxidation of glycolytic NADH via the electron transport chain, further promoting lactate accumulation [35]. Notably, recent periodontitis studies using machine learning have identified *COQ2* as a key gene and explored its mechanistic role in the disease [34].

Although *CFI* and *COQ2* were identified as key lactate metabolism‐related genes in this study, no significant enrichment of metabolism‐related pathways was observed in the GSEA results (Figure 4). This discrepancy may be explained by several factors. First, GSEA evaluates pathway enrichment based on the overall expression profile of all genes, whereas *CFI* and *COQ2* were identified through machine learning algorithms that focus on individual predictive power rather than global pathway activity [36, 37]. Second, lactate metabolism involves complex, multi‐step enzymatic reactions and regulatory networks that may not be fully captured by conventional pathway databases such as KEGG or GO. Third, the functional roles of *CFI* and *COQ2* in periodontitis may extend beyond metabolism, involving immune regulation and complement cascades, as supported by studies on immunometabolism [38, 39] and the pleiotropic nature of metabolic genes [40]. Therefore, the lack of metabolic pathway enrichment in GSEA does not contradict the identification of these genes as lactate metabolism‐related, but rather reflects the multifaceted nature of their biological functions. To further explore the functional roles of *CFI* and *COQ2*, we analysed immune cell infiltration in periodontitis. Our results revealed significant alterations in 15 types of immune cells between periodontitis and control samples, consistent with the inflammatory cascade characteristic of this disease. Notably, *CFI* showed positive correlations with neutrophils and plasma cells, while *COQ2* exhibited strong negative correlations with plasma cells, aligning with the activation of neutrophils and plasma cells in periodontitis samples, reflecting immune responses to pathogenic bacterial infection. Additionally, the expression of reparative M2 macrophages, regulatory T cells, and resting dendritic cells decreased in periodontitis groups, suggesting impaired tissue repair. *CFI* demonstrated negative correlations with M2 macrophages, regulatory T cells, and resting dendritic cells, whereas *COQ2* showed positive correlations with M2 macrophages and resting dendritic cells, consistent with these observations. We further constructed a molecular regulatory network based on the functional roles of these key genes. Future studies may investigate how *CFI* and *COQ2* regulate the immune microenvironment in periodontitis and develop novel immunomodulatory therapies targeting these genes. Based on these findings, we propose that *CFI* and *COQ2* could serve as potential targets for immunomodulatory therapy in periodontitis. Given that *CFI* is upregulated in periodontitis, strategies aimed at modulating its activity—rather than simply enhancing it—may help restore complement balance and limit excessive inflammation. In contrast, since *COQ2* is downregulated, upregulating its expression could improve mitochondrial function and reduce oxidative stress in periodontal tissues. Future studies should explore the therapeutic potential of modulating these genes using small molecules, gene editing, or biologics in preclinical periodontitis models.

Amiodarone, primarily used as an antiarrhythmic drug, has not been extensively studied or validated for its potential role in periodontitis treatment. Recent studies indicate that amiodarone inhibits the NF‐κB inflammatory signalling pathway [41] and synergizes with antibiotics to combat drug‐resistant bacteria and specific pathogens [42], suggesting its potential as an adjunct therapy for periodontitis. In this study, predictions of potential compounds targeting the key genes revealed strong binding affinity between amiodarone and CFI/COQ2, highlighting its therapeutic prospects. However, it should be noted that these findings are predictive in nature and require further experimental validation. Regarding lactate metabolism, amiodarone has been shown to elevate lactate levels in hepatocytes, likely due to its inhibition of mitochondrial electron transport system complexes I (CI) and II (CII) [35], which indirectly enhances anaerobic glycolysis and lactate accumulation. However, systemic side effects of amiodarone, such as pulmonary toxicity and cardiotoxicity [43], limit its direct application in periodontitis therapy. Future studies should focus on validating the anti‐inflammatory and osteoprotective effects of amiodarone in cellular and animal models of periodontitis, as well as developing localized delivery systems or structural derivatives to mitigate toxicity while retaining therapeutic benefits.

In vitro experiments further confirmed that the expression patterns of *CFI* and *COQ2* were consistent with our bioinformatics predictions. In the in vivo rat model of ligature‐induced periodontitis, immunohistochemical staining revealed that *CFI* expression was elevated and *COQ2* expression was reduced in periodontal tissues compared to healthy controls. Notably, both *CFI* and *COQ2* were widely expressed in multiple cell types within periodontal tissues. This finding does not contradict our correlation analysis, as bulk RNA‐seq reflects average expression across all cells and its association with immune cell proportions, rather than indicating cell type‐specific expression. Although these genes are broadly expressed in structural cells, their expression levels in specific immune cells may be dynamically regulated during periodontitis and contribute to the observed correlations. Future studies using single‐cell technologies are needed to further validate these findings.

In conclusion, this study revealed the correlation between periodontitis and lactate metabolism through bioinformatics analysis, identifying two key genes (*CFI* and *COQ2*) associated with lactate metabolism in periodontitis. Investigations into their mechanistic roles demonstrated that these genes may participate in periodontitis pathogenesis through multiple pathways and interact with immune cells, providing potential therapeutic guidance for patients. To our knowledge, this is the first bioinformatics study exploring the relationship between periodontitis and lactate‐related genes. *CFI* and *COQ2* represent the most significant crosstalk genes linking periodontitis and lactate metabolism, serving as potential diagnostic biomarkers. Overall, we propose a hypothesis that lactate‐targeted therapy or modulation of lactate‐associated biomarkers could emerge as novel strategies for periodontitis treatment.

This study has several limitations. First, while differential gene screening was performed using bioinformatics and machine learning on periodontitis versus healthy periodontal tissues, cell type‐specific gene expression variations within heterogeneous periodontal tissues were not analysed. Distinct cell populations may exhibit divergent gene expression patterns. Future single‐cell sequencing to clarify gene expression dynamics or investigations into epigenetic regulation (e.g., methylation, non‐coding RNAs) of these key genes could refine current conclusions. Second, although we validated the mRNA and protein expression of CFI and COQ2 in vitro, the in vivo validation was primarily based on semi‐quantitative immunohistochemistry in rat periodontal tissues. Therefore, these findings should be interpreted with caution and require further confirmation in human clinical samples using more quantitative methods such as RT‐qPCR or Western blot. Additionally, the datasets used may lack sufficient statistical power to generate robust evidence. Larger‐scale patient cohorts are required for external validation. Ultimately, experimental validation through cellular or animal models will be critical to confirm the biological relevance and accuracy of these findings.

## Author Contributions

**Bijun Zhu:** conceptualization, formal analysis, methodology, writing – original draft, data curation, software, visualization. **Shi Xu:** writing – review and editing, formal analysis, visualization, validation. **Mingyue Cheng:** writing – review and editing, software. **Leiying Miao:** writing – review and editing, resources, project administration, conceptualization, funding acquisition, supervision.

## Funding

This work was supported by the National Natural Science Foundation of China grant (22104054), Key Project supported by Medical Science and Technology Development Foundation, Nanjing Department of Health (ZKX24056), the Jiangsu Provincial Cadre Healthcare Program (BJ24041), and High‐Level Hospital Construction Project of Nanjing Stomatological Hospital, Affiliated Hospital of Medical School, Institute of Stomatology, Nanjing University (0024C006 and 0224C051).

## Consent

The authors have nothing to report.

## Conflicts of Interest

The authors declare no conflicts of interest.

## Supporting information


**Figure S1:** Molecular docking pose and interaction details of the candidate drug with the key genes. (A) The predicted binding pattern of the candidate drug to COQ2. (B) The predicted binding pattern of the candidate drug to CFI.

**Table S1:** The Lactic acid metabolism‐related genes.

**Table S2:** The sequences of primes.

**Table S3:** Gene set enrichment analysis for CFI.

**Table S4:** Gene set enrichment analysis for COQ2.

## Data Availability

The data that support the findings of this study are openly available in Gene Expression Omnibus (GEO) at https://www.ncbi.nlm.nih.gov/geo/, reference number GSE16134, GSE173078.
