# Supplementary material for: Identification of Key Genes Involved in Lactic Acid Metabolism in Periodontitis Based on Bioinformatics Analysis
Source: J Cell Mol Med. 2026 Apr 17;30(8):e71141. doi: 10.1111/jcmm.71141 (PMC13090170; doi:10.1111/jcmm.71141)
Supplement: Supplementary file 1 — Figure S1: Molecular docking pose and interaction details of the candidate drug with the key genes. (A) The predicted binding pattern of the candidate drug to COQ2. (B) The predicted binding pattern of the candidate drug to CFI. [file JCMM-30-e71141-s001.docx]

**
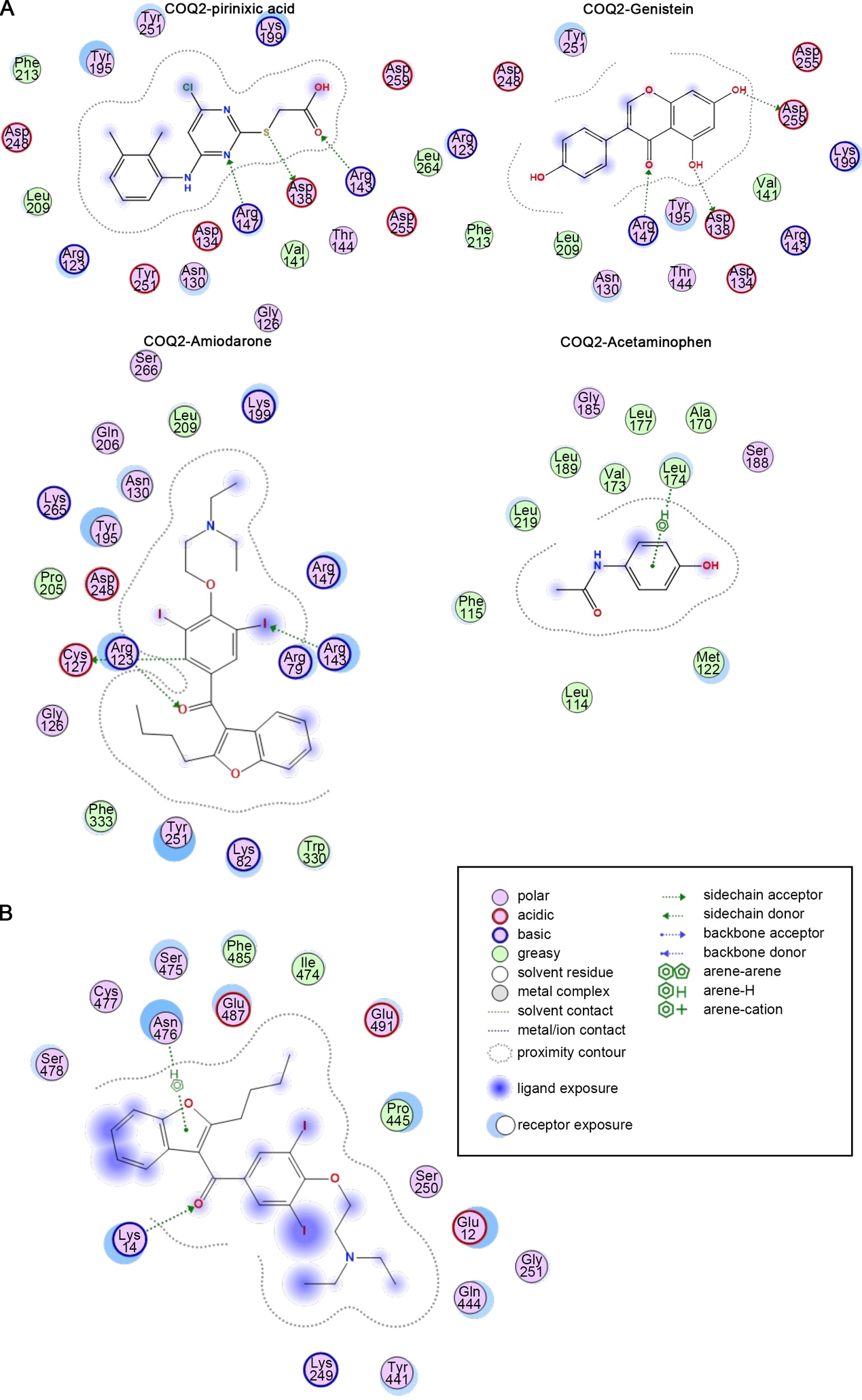
**

**Fig. S1 Molecular docking pose and interaction details of the candidate drug with the key genes.** (**A**) The predicted binding pattern of the candidate drug to COQ2. (**B**) The predicted binding pattern of the candidate drug to CFI.
