# Supplementary material for: Identification of Key Genes Involved in Lactic Acid Metabolism in Periodontitis Based on Bioinformatics Analysis
Source: J Cell Mol Med. 2026 Apr 17;30(8):e71141. doi: 10.1111/jcmm.71141 (PMC13090170; doi:10.1111/jcmm.71141)
Supplement: Supplementary file 3 — Table S2: The sequences of primes. [file JCMM-30-e71141-s005.docx]

**Table S2 The sequences of primes.**

| Gene | Forward primer sequence (5’-3’) | Reverse primer sequence (5’-3’) |
| --- | --- | --- |
| IL-1β | TGATGGCTTATTACAGTGGC | TGTAGTGGTGGTCGGAGATT |
| IL-6 | GGTGTTGCCTGCTGCCTTCC | GTTCTGAAGAGGTGAGTGGCTGTC |
| IL-8 | ACTCCAAACCTTTCCACC | CTTCTCCACAACCCTCTG |
| TNF-α | CGTGGAGCTGGCCGAGGAG | AGGAAGGAGAAGAGGCTGAGGAAC |
| RUNX-2 | GGAGTGGACGAGGCAAGAGTTT | AGCTTCTGTCTGTGCCTTCTGG |
| OCN | GGCAGCGAGGTAGTGAAGAG | GATGTGGTCAGCCAACTCGT |
| OPN | TGCTACAGACGAGGACATCACC | TCTGGACTGCTTGTGGCTGTG |
| CFI | GGAAACGAATTGTGGGAGGAA | GTGCAGCAGTCAGAATCCAAC |
| COQ2 | AAGAACAGCCAATCGTCCAAT | GGCTAGTTGAGGCCAGTATGAAA |
| GAPDH | CGCTCTCTGCTCCTCCTGTT | CCATGGTGTCTGAGCGATGT |
